# Comparative Evaluation of Stress Distribution and Permeability Characteristics in Bentonite Cutoff Walls Using CPTU and ABAQUS Methods

**DOI:** 10.3390/ma18163919

**Published:** 2025-08-21

**Authors:** Xuepeng Li, Yufu Li, Chao Yan, Fengyun Wang, Xiaoyan Liu

**Affiliations:** 1 School of Civil Engineering, Guangdong Construction Polytechnic, Qingyuan 511500, China; lixuepeng1984@126.com (X.L.); liyufu@gdcvi.edu.cn (Y.L.); 2 Anhui Institute of Intelligent Underground Detection Technology, Anhui Jianzhu University, Hefei 230601, China; yanchao729@ahjzu.edu.cn (C.Y.); wangfy@ahjzu.edu.cn (F.W.); 3 School of Mechanics and Civil Engineering, China University of Mining and Technology, Xuzhou 221116, China

**Keywords:** bentonite, cutoff wall, permeability, lateral effective stress, CPTU, ABAQUS

## Abstract

Bentonite materials are extensively used in cutoff walls at landfill sites. This study calculates the stress and permeability characteristics of bentonite materials using the piezocone penetration test (CPTU) and ABAQUS simulations. The lateral effective stress of bentonite materials is evaluated using arching models, lateral squeezing models, and a modified lateral squeezing model. Pore pressure dissipation types are categorized into standard and non-standard, with the coefficient of consolidation obtained using the half dissipation time of excess pore pressure (*t*_50_) method. In the standard dissipation type, the excess pore pressure gradually dissipates over time after the cone stops penetrating. In contrast, the non-standard dissipation type is characterized by an initial increase in pore pressure until it reaches a maximum value, followed by a decrease to hydrostatic pressure. Additionally, the pore pressure dissipation process in bentonite cutoff walls is recorded and analyzed over various time intervals. Finally, the relationship between hydraulic conductivity and *t*_50_ at landfill sites is established based on standard and non-standard dissipation types using CPTU and ABAQUS methods. The *t*_50_ method is used for the standard dissipation type, while a modified *t*_50m_ method is used for the non-standard dissipation type from CPTU and a *t*_50m_ method is used in the non-standard dissipation type from CPTU. The *t*_50m_ is the modified value derived from *t*_50_. Cutoff walls made from bentonite materials offer the advantage of enhancing the isolation effects and meeting the design requirement of permeability (1.0 × 10^−7^ cm/s).

## 1. Introduction

The most commonly used isolation and anti-seepage systems in landfills are horizontal anti-seepage systems and vertical cutoff walls, which can be constructed using curtain grouting around the landfill [1]. Techniques such as bentonite and curtain grouting for establishing vertical cutoff walls around landfills are noted for their effective anti-seepage effect, low cost, and ease of repair, indicating a broad range of application possibilities [2].

The materials for cutoff walls primarily consist of a mixture of dry bentonite, soil, and bentonite slurry [3]. Soil–bentonite cutoff walls offer lower permeability and better uniformity compared to those made from topsoil or cement-bentonite. From the in situ studies, the bentonite content in cutoff wall material ranges from 4% to 8%, with a solid content of approximately 70% [4]. D’Appolonia [5] suggested that the thickness of the cutoff wall should be between 0.6 m and 1.5 m, considering the size of the slotting machine.

The piezocone penetration test (CPTU) is a newly developed in situ testing method for evaluating the coefficient of consolidation and hydraulic conductivity during the dissipation process [6,7,8]. Bentonite-based cutoff walls are widely used in polluted site remediation due to their simple construction and robust anti-seepage properties [9,10,11]. CPTU technology combines traditional static cone penetration testing with pore pressure element testing [12]. Lunne et al. [13] summarized most current piezocone penetration test methods for predicting permeability, highlighting the importance of the coefficient of consolidation. Sully et al. [14] applied CPTU technology to overconsolidated soil and proposed the *t*_50m_ method for evaluating the parameters of overconsolidated soil. Elsworth and Lee [15] introduced a technique for assessing soil permeability using CPTU technology. Chai et al. [16] developed a new method for calculating consolidation permeability through numerical simulations. Jiang et al. [17] optimized the sodium hexametaphosphate content in the cutoff wall to 2% for improved workability.

Dong et al. [18] conducted a geotechnical centrifuge model test to illustrate the cumulative impact, which increased with depth and reached its maximum value. To investigate the uncertainty of the stress state, Evans et al. [19] were the first to study it and concluded that the sidewall frictional resistance of the cutoff wall caused a stress distribution within the cutoff wall material. The effective vertical stress can be expressed by Equation (1).

(1) $${\sigma_{v}}^{\prime }=\frac{\frac{B}{2}\left(\gamma_{b}^{\prime }-2\frac{c_{b}}{B}\right)}{k_{b}tan\phi_{b}^{\prime }}\left(1-exp\left(-2k_{b}\frac{z}{B}tan\phi_{b}^{\prime }\right)\right)$$

where *B* is the thickness of the cutoff wall, $\gamma_{b}^{\prime }$ is the effective density, *k*_b_ is the coefficient of Earth pressure at rest, *c*_b_ is the internal cohesion, *δ* is the internal friction angle, *k*_b_ is the cohesion, $\phi_{b}^{\prime }$ is the effective internal friction angle, and *z* is the calculated depth.

Xu et al. [20] utilized ABAQUS software to further develop the Duncan–Chang constitutive model. Shi et al. [21] employed Fast Lagrangian Analysis of Continua in 3 Dimensions (FLAC3D) for soil consolidation and simulated engineering cases, with the simulation values showing agreement with the measured values. Sun and Gao [22] used ABAQUS analysis to simulate the soil consolidation process. Chai et al. [23] simulated the penetration process of a CPTU probe with ABAQUS and provided a cloud map to depict the spatial distribution of induced pore pressure over time.

The CPTU empirical formula is primarily applicable to cohesive soil, and relatively little research has focused on soil–bentonite materials. These formulas may not be suitable for soil–bentonite. Further efforts are needed to increase the application of CPTU technology in soil–bentonite cutoff walls, providing reference value for evaluating the physical and mechanical characteristics of bentonite cutoff walls using CPTU technology.

The purpose of this experiment is to study the effective stress distribution of a bentonite cutoff wall, as derived from three theoretical models. Additionally, it aims to establish the relationship between the *c*_h_ and the modified *t*_50_, as determined by CPTU for two different pore pressure dissipation types. Considering that ABAQUS can provide a cloud map to depict the distribution of induced pore pressure over time, this paper also uses ABAQUS to investigate the pore pressure of bentonite cutoff walls at various times.

## 2. Materials and Methods

### 2.1. Test Materials

A test project for this study was conducted at a landfill site in China. The bentonite used for the cutoff wall was hydrogel from the Wyo-Ben, Inc., Greybull, WY, USA, while the base soil was sourced from the topsoil between 0 and 1 m below the surface. The materials consisted of 5% bentonite and 95% backfill soil by weight.

The liquid limit of the bentonite was 281.2%, the plastic limit was 64.1%, and the plasticity index was 217.1%. According to the ASTM D2487 [24] classification, bentonite is classified as high plastic clay (CH), positioned above line A and to the right of line B on the plasticity chart. In contrast, the liquid limit of the backfill soil is 30.1%, the plastic limit is 19.6%, and the plasticity index is 10.5%. This classifies the backfill soil as low plastic clay (CL), positioned above line A and to the left of line B on the plasticity chart.

**Figure 1:**
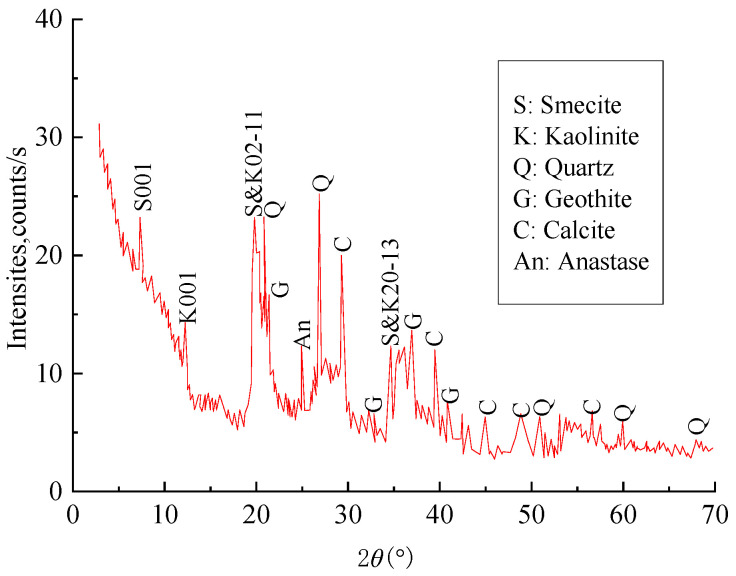
The XRD of bentonite.

Figure 1 presents the XRD results of the bentonite used in the experiment. The XRD analysis indicates that the clay minerals present include montmorillonite (S) and kaolinite (K), along with non-clay minerals such as quartz (Q), calcite (C), goethite (G), and rutile (An). The figure shows that bentonite comprises 84% clay minerals and 16% non-clay minerals. Montmorillonite constitutes 46% and kaolinite 38% of the clay mineral composition. Among the non-clay minerals, quartz accounts for 3.1%, rutile 0.9%, calcite 3.6%, and goethite 8.4%

### 2.2. CPTU

**Figure 2:**
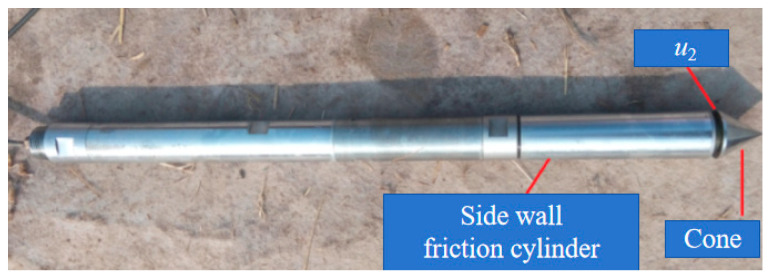
The piezocone used in this study.

The test was conducted by the piezocone penetration test system imported from Hogentogler, Columbia, MD, USA, equipped with the latest multifunctional digital probe. The system comprises two main components: a drilling vehicle and a static probe system, both of which are equipped with four functional digital hole pressure probes. The system includes several conventional functions, as well as a coneplot and cleanup data processing software 2.0. The CPTU technology not only measures pore pressure but also monitors the dissipation process of excess pore pressure caused by penetration below the groundwater level (Figure 2).

### 2.3. ABAQUS

To simulate the penetration process of soil–bentonite cutoff walls using ABAQUS 6.14, the model has a thickness of 10 m and a width of 4.6 m. The soil–bentonite cutoff wall is positioned in the center of the model and has a width of 0.6 m. The bottom of the soil layer is undrained, while the top surface is drained. Prior to loading, the soil has consolidated under its own weight stress. Subsequently, a CPTU penetration test is conducted in the soil layer, with the probe reaching a depth of 5 m. The groundwater level is located at a depth of 1 m below the soil–bentonite cutoff wall. This calculation example utilizes the Modified Cambridge Model [25], with the following model parameters: slope *K* is 0.01, slope *λ* is 0.1, slope *M* is 1.2, Poisson’s ratio *ν* is 0.33, static side pressure coefficient *k* is 0.56, the soil saturation bulk density is 17.7 kN/m^3^, the water bulk density is 10 kN/m^3^, and the permeability coefficient is 4.1 × 10^−8^ cm/s. The model parameters are detailed in Table 1 [26,27,28,29].

Equation (2) represents the yield equation of the Modified Cambridge Model [25]. Since the yield trajectory resembles an elliptical curve, it more accurately reflects the characteristics of soil deformation compared to the Cambridge model.

(2) $$\left(1+\frac{q^{2}}{M^{2}p^{2}}\right)p=p_{0}$$

where *p* is the ball stress, also known as the average normal stress, *q* is the deviatoric stress, *p*_a_ is the initial stress, and *M* is the slope of the critical state line.

ABAQUS is employed to simulate the penetration and consolidation process. The initial conditions of the model must be established in accordance with the actual conditions of the cutoff wall (Table 2). To achieve the most accurate calculation results, it is essential to utilize the most precise initial conditions. Specifically, the initial effective stress at the top surface of the soil layer (*y* = 10 m) is *σ_x_*′ = 0 kPa and *σ_y_*′ = 0 kPa, while that at the bottom surface (*y* = 0 m) is *σ*_x_′ = 38.5 kPa and *σ_y_*′ = −77 kPa. The initial pore pressure *u* at the top surface of the soil layer is 0 kPa, whereas at the bottom of the soil layer, it is *u* = 90 kPa.

For the soil–bentonite mixture, the initial porosity ratio (*e*_0_) at the top of the soil layer (*y* = 10 m) is 0.96, compared to 0.54 at the bottom (*y* = 0 m). In the outer foundation soil of the soil–bentonite wall, the *e*_0_ value at the top (*y* = 10 m) is 0.79, and at the bottom (*y* = 0 m), it is 0.51. The initial dry density at the upper section of the soil layer (*y* = 10 m) is 1.28 g/cm^3^, while at the lower section (*y* = 0 m), it is 1.42 g/cm^3^. The initial yield surface size at the top of the soil layer (*y* = 10 m) is *p*′ = 0 kPa, *q* = 0 kPa, and *p*_0_′ = 0 kPa, and at the bottom (*y* = 0 m), the values are *p*′ = 51 kPa, *q* = 38.5 kPa, and *p*_0_′ = 80 kPa.

## 3. Results and Analysis

### 3.1. Lateral Effective Stress

**Figure 3:**
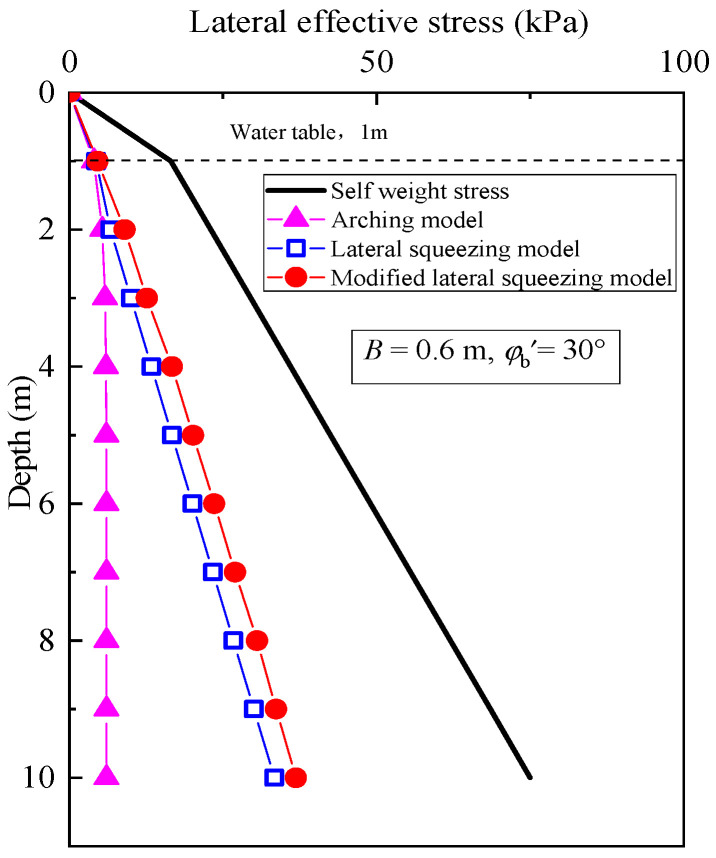
Lateral effective stress distribution based on different theoretical models.

The evaluation of lateral effective stress of the cutoff wall at the landfill site using various theoretical models is presented in Figure 3. The cutoff wall has a width of 1.2 m, a cohesion of 0 kPa, and an effective internal friction angle 30°. The assessment of lateral effective stress is based on the arching model, the lateral squeezing model [29], and the modified lateral squeezing model [30]. The lateral effective stress can be expressed as

(3) $$\sigma_{h}^{\prime }=\frac{2{\Delta D}_{b}}{B}$$

where $\sigma_{h}^{\prime }$ is lateral effective stresses of the wall, $k_{am}$ is the coefficient of lateral pressure of soil, Δ is the lateral deformation of the wall, and $D_{b}$ is the lateral modulus [29].

(4) $$\sigma_{h}^{\prime }=10^{\left(\frac{2\Delta -BC_{1}}{BC_{c\varepsilon }}\right)}$$

where *C*_1_ is the strain value under unit stress of the wall and *C*_cε_ is the modified compression index of the wall [30].

This study reveals that the lateral effective stress values obtained from all three models are lower than those resulting from self-weight stress, particularly at greater depths. Moreover, the results generated by the arching model are lower than those produced by the other two models. However, when a rigid cutoff wall is considered, the arching model may underestimate the actual stress distribution.

### 3.2. CPTU Results

**Figure 4:**
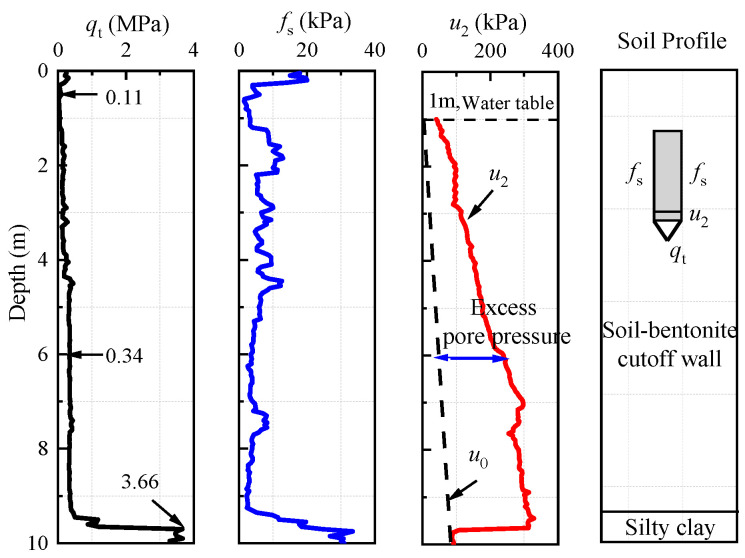
The typical test curve of the soil–bentonite cutoff wall.

Figure 4 presents the typical test results and soil layer distribution as determined by CPTU. The figure indicates that the cone tip resistance (*q*_t_) is 0.11 MPa at 0.5 m and increases to 0.34 MPa at 6 m. The pore pressure exceeds static pore pressure (*u*_0_), with this excess attributed to penetration of the probe. The classification of the soil layers reveals that the soil is relatively soft and exhibits high pore pressure, thus categorizing it as a soil–bentonite layer. At approximately 9.3 m of depth, the cone tip resistance shows a significant increase, peaking at around 3.66 MPa. Correspondingly, the pore water pressure (*u*_2_) significantly decreases to a level comparable to static pore pressure. The soil at this depth is relatively hard with minimal or no pore pressure, indicating that the probe has penetrated into the original soil layer, which can be classified as a silty clay layer.

### 3.3. Pore Pressure Dissipation Types

The types of pore pressure dissipation are categorized into standard and non-standard pore pressure dissipation types, as illustrated in Table 3. Excess pore pressure dissipates over time, ultimately reaching 0 kPa, a process referred to as the standard dissipation type. In contrast, in the non-standard dissipation type, the excess pore pressure resulting from probe penetration initially rises to a maximum before starting to decrease. To evaluate the *c*_h_ for the standard dissipation type, the *t*_50_ method is applicable. However, for the non-standard dissipation type, the *t*_50_ values must be adjusted. This study employs a modified *t*_50m_ method instead of *t*_50_ to obtain calculation results that align more closely with engineering realities.

The primary factors contributing to the observed variations in dissipation on-site are as follows: Initially, the pore pressure sensor lacks full saturation. As water permeates the sensor, pore pressure steadily increases until complete saturation is achieved. Another crucial factor is the inconsistent mixing of the soil–bentonite material, which can cause shear dilation in specific regions, leading to pore pressure redistribution. The pattern resulting from shear dilation differs across various locations on the cone. For instance, the pore pressure at the cone shoulder and other conical surfaces is lower than that in the surrounding soil. During the consolidation phase, the elevated excess pore pressure in the adjacent soil is redistributed, leading to an increase in excess pore pressure on the cone surface [31,32].

When evaluating the consolidation coefficient, determining the time *t*_50_ corresponding to 50% dissipation of excess pore pressure is crucial. A standard dissipation type of pore pressure is observed at 5.35 m, while a non-standard dissipation type occurs at 7.10 m for the soil–bentonite cutoff wall, as shown in Table 3. Half of the excess pore pressure, Δ*u*_i_/2, is 9.05 kPa, corresponding to a *t*_50_ of 810 s. The maximum values resulting from probe penetration at 7.10 m are 16.4 kPa, with a dissipation time *t*_50_ of 305 s.

### 3.4. Coefficient of Consolidation and Permeability

**Figure 5:**
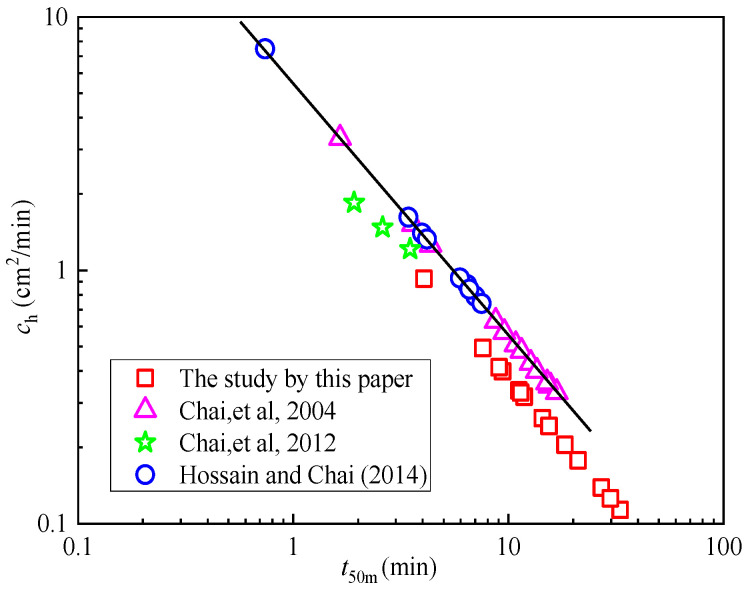
The *c*_h_ values based on the modified *t*_50m_ method by CPTU [23,33,34].

The relationships between the *c*_h_ values and *t*_50m_ is illustrated in Figure 5. The *t*_50m_ values range from 9.4 to 33.1 min, while the *c*_h_ values vary between 0.11 and 0.96 cm^2^/min. Additionally, Figure 5 presents the results of the numerical analysis conducted by Chai et al. [23,33], which report *t*_50m_ values ranging from 1.65 to 16.78 min and the *c*_h_ values ranging from 0.33 to 3.33 cm^2^/min. Chai et al. [23,33] focused their research on overconsolidated clay, whereas the materials examined in this paper are in an underconsolidated or normally consolidated state. The variation in *c*_h_ and logarithmic time investigated in this paper is relatively similar to those observed in previous experiments [34].

**Figure 6:**
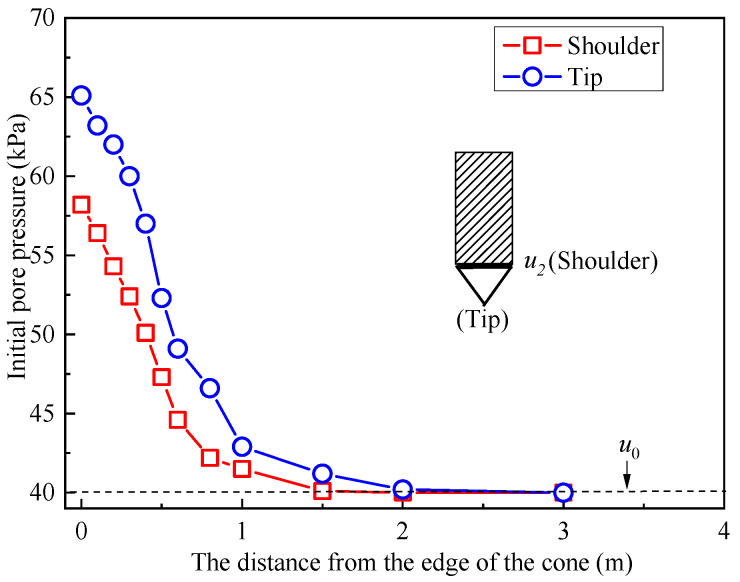
The initial pore pressure of soil–bentonite from CPTU cone (5 m).

Figure 6 demonstrates the pore pressure distribution surrounding the CPTU cone, emphasizing the pressures at the cone’s shoulder and tip. The figure reveals that, at an equal horizontal distance from the cone’s edge, the pore pressure at the tip exceeds that at the shoulder. Furthermore, the initial pore pressure distribution can be categorized into two stages [35]. Near the cone’s edge, as the distance increases, the initial pore pressure experiences a sharp decline. In contrast, at a greater distance from the probe, the initial pore pressure decreases more gradually, eventually approaching hydrostatic pressure.

**Figure 7:**
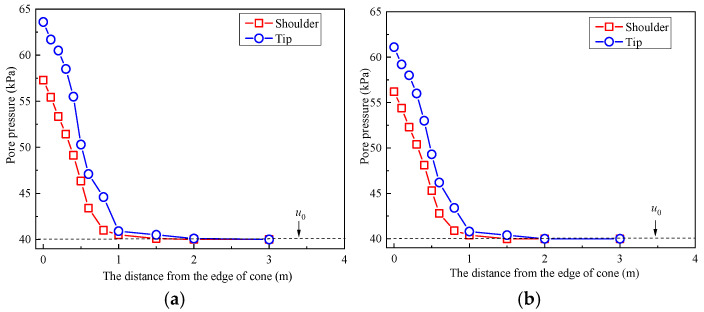

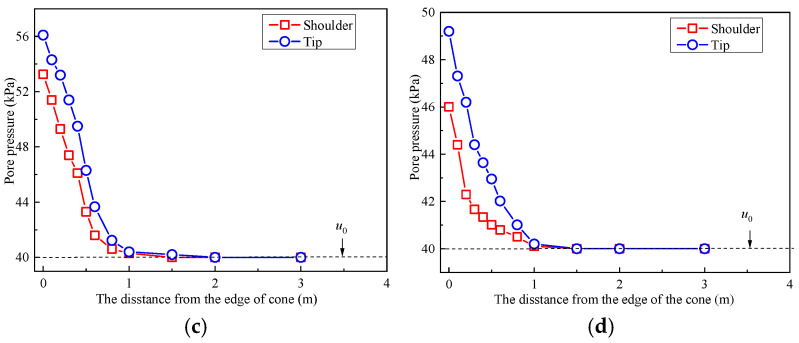
The pore pressure of mixtures from the CPTU cone (5 m). (**a**) Dissipated time: 5 s; (**b**) dissipated time: 30 s; (**c**) dissipated time: 180 s; (**d**) dissipated time: 1800 s.

The dissipation rate of the cutoff wall is related to the permeability coefficient. Figure 7 displays the pore pressure of the soil–bentonite mixtures at various times, measured at 5 m below the surface. The figure is divided into four smaller graphs, each corresponding to different dissipation times: 5 s, 30 s, 180 s, and 1800 s. Figure 7 indicates that the pore pressure values are inversely proportional to the distance from the cone’s edge. As the radial distance increases to a certain extent, the pore pressure gradually approaches or equals to *u*_0_. Additionally, at the same radial distance, the pore pressure at the cone tip is slightly higher than that at the cone shoulder. Notably, when the radial distance is approximately 1 m, the pore pressure during all four time intervals is essentially equal to the hydrostatic pressure [36].

**Figure 8:**
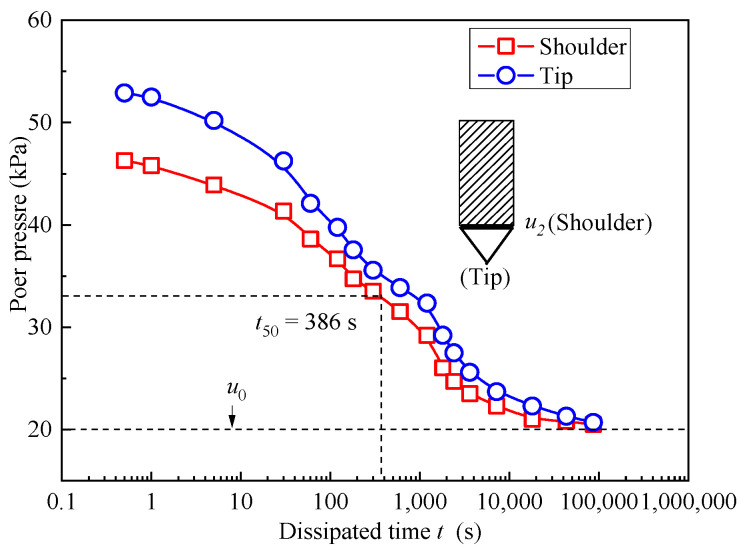
The pore pressure dissipation curves for soil–bentonite mixtures at various locations at a depth of 3 m.

Figure 8 illustrates significant variations in pore pressure curves for soil–bentonite mixtures at different locations, as simulated by ABAQUS at a depth of 3 m. The figure indicates that the initial pore pressures at the cone’s shoulder and tip are 46.5 kPa and 53.5 kPa, respectively, at this depth. Over time, the pore pressure dissipates gradually [37]. At the onset of dissipation, the pore pressure drops sharply and as dissipation time increases, the rate of decrease in pore pressure slows down. The time required for half of the excess pore pressure to dissipate at the cone’s shoulder is 13.25 kPa. At this point, the pore pressure is 33.25 kPa, with the corresponding *t*_50_ being 386 s. In the CPTU test, the *t*_50_ value at 3 m was determined to be 405 s based on pore pressure dissipation.

**Figure 9:**
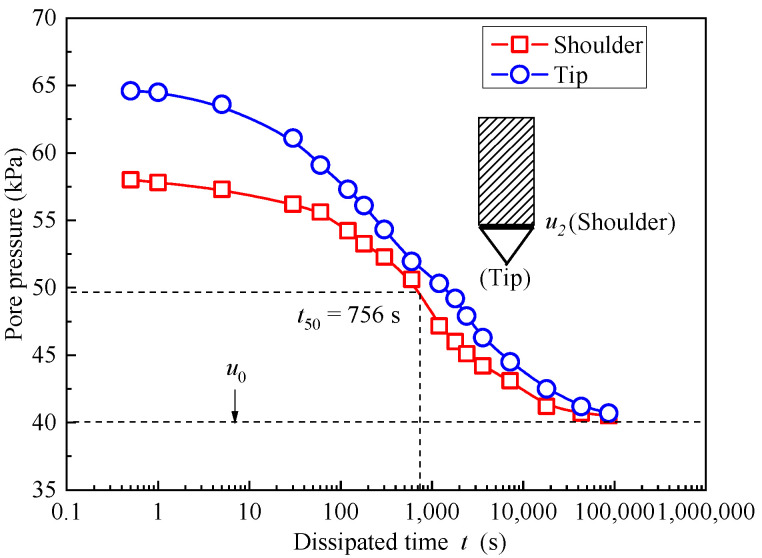
The dissipation curves of soil–bentonite materials based on ABAQUS with a depth of 5 m underground.

The relationship between pore pressure values and dissipation time (Figure 9) shows significant variations at a depth of 5 m. It is evident that the initial pore water pressures at the cone’s shoulder and tip are 58.1 kPa and 65.1 kPa, respectively, at a depth of 3 m. At the cone’s shoulder, the time required for half of the excess pore pressure to dissipate is 9.05 kPa. At this point, the pore pressure value is 49.05 kPa, with a corresponding *t*_50_ of 756 s. During the CPTU field test, the *t*_50_ value at 5 m was found to be 810 s, determined by pore pressure dissipation. Therefore, when comparing the *t*_50_ values obtained from the CPTU field testing results and numerical simulations at both 3 m and 5 m, it is clear that the values from the two distinct methods are quite similar.

**Figure 10:**
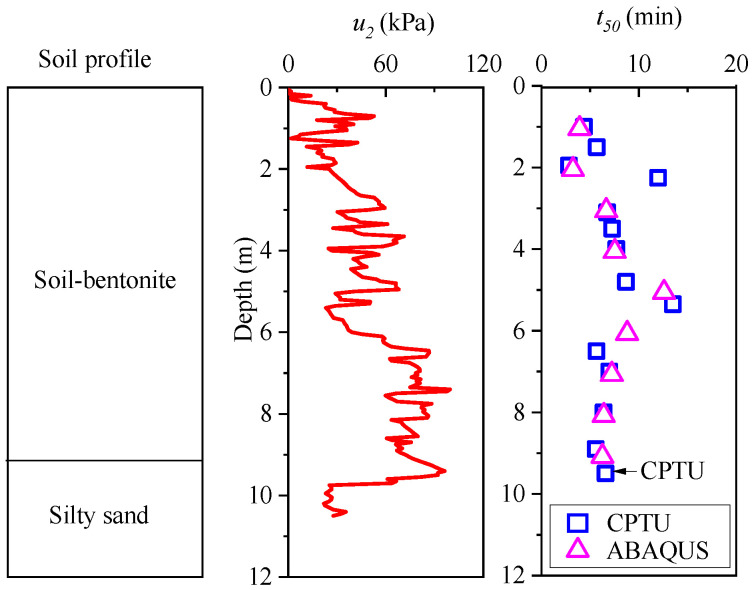
The *t*_50_ values at different depths by CPTU and ABAQUS.

Using the aforementioned method, pore pressure dissipation curves and *t*_50_ values at various depths can be derived. Figure 10 illustrates the measured *u*_2_ and *t*_50_ values along the depth of the wall. The *u*_2_ values decrease sharply when the cone penetrates into the silty sand layer. Additionally, a comparison between the CPTU data and the ABAQUS simulation results reveals a strong correlation between the *t*_50_ values obtained from CPTU and those simulated by ABAQUS. This suggests that the numerical model employed in ABAQUS effectively captures the soil response under CPTU conditions.

**Figure 11:**
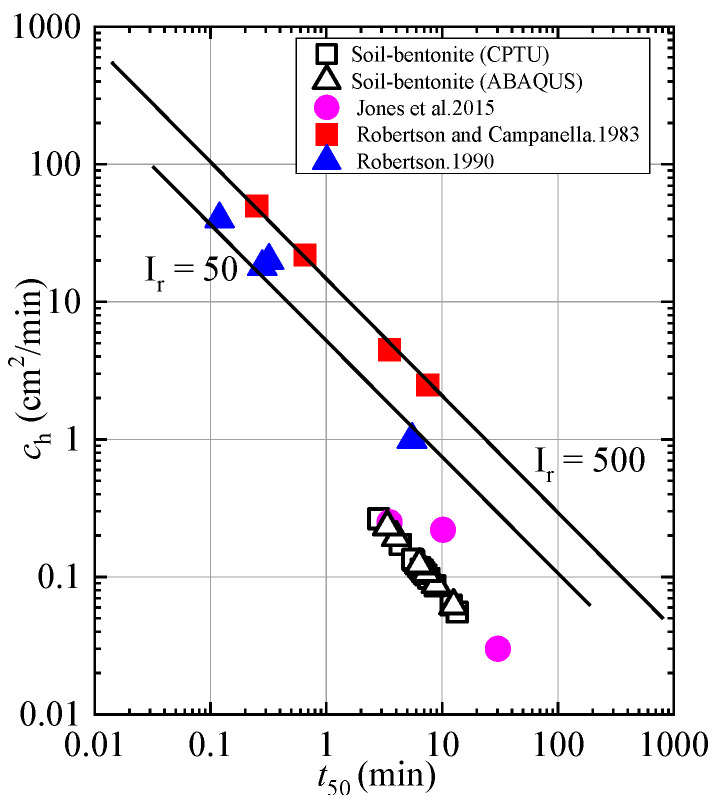
Comparative analysis of coefficient of consolidation based on different methods [38,39,40].

Figure 11 presents a comparative analysis of the *c*_h_ values derived from various methods and other studies [38,39,40]. The figure illustrates the relationship between *c*_h_ values and *t*_50_ values. Given the similarity between ABAQUS simulations and CPTU results, the *t*_50_ values obtained from both methods are quite comparable, and consequently, the coefficient of consolidation values are also similar. It is evident that the *c*_h_ values for the soil–bentonite cutoff wall range from 0.06 to 0.23 cm^2^/min based on numerical simulations, whereas the value according to CPTU range from 0.056 to 0.265 cm^2^/min. The relationship between the coefficient of consolidation and *t*_50_ studied in this paper differs slightly from that in the cited literature [38,39,40], primarily because the materials in the cited literature consist of cohesive soil, while this paper focuses on a soil–bentonite mixture. The results are quite similar to those reported by Jones et al. (2015) [38].

The link between the permeability coefficient *k*_h_ and *t*_50_ using various methods is demonstrated in Table 4. The table presents the results of both non-standard and standard dissipation based on CPTU testing at the same depth, alongside the results from Robertson et al. [41] for the permeability and *t*_50_. Multiple methods indicate that the permeability is lower than the design standard (1.0 × 10^−7^ cm/s), thereby meeting the isolation requirement.

## 4. Conclusions

In this study, a cutoff wall composed of soil-mixed bentonite was analyzed for a landfill site using CPTU and ABAQUS methods. The aim of this paper is to evaluate the lateral effective stress and permeability of the bentonite material cutoff wall. Stress is assessed using three models. The relationship between hydraulic conductivity and *t*_50_ at a landfill site is derived from both standard and non-standard dissipation types. The main conclusions of this paper are as follows:

1. The lateral effective stress of the soil–bentonite mixtures, determined by the arching model, is lower than that calculated using the lateral squeezing model and the modified lateral squeezing model.
2. The *t*_50_ parameter is crucial for evaluating *c*_h_ and *k*_h_. This paper provides the *t*_50_ values at various depths using the CPTU method. The *t*_50_ value is 810 s at 5.35 m for the standard dissipation type and 305 s at 7.10 m for the non-dissipation type. The *t*_50_ values obtained from CPTU are relatively close to those simulated by ABAQUS.
3. The *k*_h_ values (<1.0 × 10^−7^ cm/s) of the materials used, obtained through various methods, indicate that they meet the isolation requirements when compared with previous research results.

## Figures and Tables

**Table 1:** The parameters of the Modified Cambridge Model [25].

Parameter	Size	Units	Reference
*K*	0.01	/	[26]
*λ*	0.1	/	[26]
*M*	1.2	/	[26]
*ν*	0.33	/	[27]
*k_0_*	0.56	/	[26]
*γ_rat_*	17.7	kN/m^3^	[27]
*γ_w_*	10	kN/m^3^	[28]
*k_h_*	4.1 × 10^−8^	cm/s	[28,29]

**Table 2:** The initial conditions of the Modified Cambridge Model [25].

Parameter	Value	Unit
Initial effective stress	*y* = 10 m: σ_x_′ = 0, σ_y_′ = 0 *y* = 0 m: σ_x_′ = 38.5, σ_y_′ = −77	kPa
Initial pore pressure	*y* = 10 m: *u* = 0 *y* = 0 m: *u* = 90	kPa
Initial pore pressure	*y* = 10 m: *e*_0_ = 0.96 *y* = 0 m: *e*_0_ = 0.54	/
Initial dry density	*y* = 10 m: *ρ*_d_ = 1.28 *y* = 0 m: *ρ*_d_ = 1.42	g/cm^3^
Initial yield surface	*y* = 10 m: *p*′ = 0, *q* = 0, *p*_0_′ = 0 *y* = 0 m: *p*′ = 51, *q* = 38.5, *p*_0_′ = 80	kPa

**Table 3:** The caused pore pressure and dissipation time of two different types.

CPTU Types	Δ*u_i_* (kPa)	Δ*u_i_*/2 (kPa)	Δ*u_max_* (kPa)	*t*_50_ (s)
Standard dissipation	18.1	9.05	/	810
Non-Standard dissipation	5.49	/	16.4	305

**Table 4:** The *k*_h_ and *t*_50_ values based on different methods.

Parameter	Standard Dissipation	Non-Standard Dissipation	ABAQUS	Robertson et al., 1993 [41]
*t*_50_ (min)	12.0	11.2	12.1	12.6
*k*_h_ (cm/s)	9.3 × 10^−9^	2.7 × 10^−8^	8.5 × 10^−8^	1.2 × 10^−8^

## Data Availability

The original contributions presented in this study are included in the article. Further inquiries can be directed to the corresponding author.
